# American Association of Obstetricians and Gynecologists

**Published:** 1896-09

**Authors:** 


### MEDICAL ITEMS.

Dr. A. B. Holder, a well known surgeon of Memphis, died July 13th of acute appendicitis.

Dr. R. W. Fort has moved from Atlanta to Bainbridge, where he will be physician to a large convict camp. Our good wishes go with him.

The next annual meeting of the Tri-State (Alabama, Georgia, and Tennessee) Medical Society will be held at Chattanooga, Tenn., on October 13, 14, and 15, 1896. Dr. I. B. Murfree, Murfreesboro, Tenn., is President, and Dr. F. Trester Smith, Chattanooga, is Secretary.

Picric acid, a drachm to two ounces of alcohol and twenty of water, is highly recommended as an application by compresses to burns. The solution is commended as being soothing, antiseptic, and non-poisonous. Two or three applications at intervals of several days are usually sufficient.—*Denver Med. Times*, June.

Great caution must be observed in the use of cocaine within the urinary passages, says a writer in *Therapeutische Wochenschrift,* June 21, but the capricious action of the drug is as difficult to guard against as that of chloroform. The use of cocaine is contraindicated in anemic persons and in those that are the subjects of respiratory or circulatory disease. When cocaine poisoning occurs, amyl nitrite and chloroform should be used, also opium and chloral hydrate for the convulsions, but above all artificial respiration and injections of camphor dissolved in ether.

MR. ALVIN ASHER.—We regret to announce the death of young Mr. C. A. Asher. He was a student of the Atlanta Medical College, in his third term. Always happy and good-natured, he will be sadly missed by those who knew him.

---

RUBBER medical and surgical goods are said to rot so readily because of the action of the atmosphere on the rubber, changing the sulphur in it into sulphuric acid. Such goods can be preserved for a much longer period by occasional immersion in an alkali fluid.—*Denver Med. Times*, June.

---

*The Alabama Medical and Surgical Age* states that at the meeting in April next of the Medical Association of the State of Alabama, which was founded by Dr. Jerome Cochran, a proposition will be brought forward to commemorate Dr. Cochran's unselfish and philanthropic work by raising a fund to build in Selma a monument to his memory.

---

A DEATH CERTIFICATE.—This unique specimen of a certificate of death is said by an English contemporary to have been tendered by a native apothecary at an inquest in India: "I think she died or lost her life for want of food, or on account of starvation, and perhaps for other things of her comfortables, and most probably she died from drowning."—*Medical Record.*

---

We desire to remind our readers that the twentieth annual meeting of the American Dermatological Association will be held at the Hot Sulphur Springs of Virginia, September 8, 9, and 10, 1896. Officers for 1896: President, A. R. Robinson, M.D., of New York; Vice-President, F. J. Shepherd, M.D., of Montreal; Secretary and Treasurer, C. W. Allen, M.D., 126 East Sixtieth street, New York.

THE sixth annual meeting of the American Electro-Therapeutic Association will be held on Tuesday and Wednesday, September 29th and 30th, and Thursday, October 1, 1896, in Allston Hall, the Studio Building, on Clarendon Street, near St. James avenue, Boston, Mass. The Brunswick Hotel, corner Boylston and Clarendon streets, will be the headquarters of the Association during the meeting. Terms reduced to four dollars per day, for Fellows and their friends. The Copely Square Hotel, corner Exeter street and Huntington avenue, terms reduced to three dollars per day, is also located in close proximity. For rooms, etc., apply either to the proprietors, or to Dr. W. H. White, No. 222 Marlborough street, Boston, Mass., who, as the Vice-Chairman of the Committee of Arrangements, will reserve rooms.

---

The *Lancet*, for June 27th, contains an account of a case of uterine hemorrhage, bringing the patient to the verge of death, which was, however, fortunately averted by the injection of saline fluid into the axillary cellular tissue. The credit for this method of treatment is due to Mr. Thomas B. Morse, of Norwich. The instrument employed is a slight modification of Mr. Arbuthnot Lane's transfusion apparatus, and an ordinary saline solution is used—a teaspoonful of common salt to the pint of boiled water' cooled down to 100° F. The steel point of the needle is thrust through the skin of the axilla deep enough to move freely in the cellular tissue, which is slowly filled with the solution, the syringe being refilled until a pint or more has been injected.

In this case the fluid was rapidly absorbed, and hardly any swelling remained at the end of two hours. The patient rapidly showed signs of improvement, and was soon able to take nourishment.

The advantages of this method are obvious, for any physician could use the instrument unassisted, and with little loss of time.

THE following law relative to ophthalmia neonatorum is now in operation in South Carolina. There should be something similar in every State: "Be it enacted by the General Assembly of the State of South Carolina, that should one or both eyes of an infant become reddened or inflamed at any time after birth, it shall be the duty of the midwife or nurse or person having charge of said infant to report the condition of the eyes at once to the local board of health of the city or town in which the parents of the infant reside; that the Secretary of State shall cause a sufficient number of copies of this act to be printed, and supply the same to health officers and health committees, whose duty it shall be to furnish a copy to each person who is known to act as midwife or nurse in the cities or towns for which they have been appointed; any failure to comply with the provisions of this act shall be punishable by a fine not to exceed $25, or imprisonment not to exceed one month, or both."

---

DR. LOVE announces in the *Medical News* his retirement from the staff of the Marion-Sims College of Medicine. His resignation was accepted, accompanied by expressions of appreciation and good wishes.

Dr. Love took advantage of the occasion of this announcement to state in the *Medical News* that "there are about four times as many medical colleges in every section of the country as are needed. . . . Men are holding professorships to-day in medical colleges who, figuratively speaking, are still in diapers and who wear their honors and perform their duties about as gracefully as the teething infant handles his rubber ring and his rattle. The late Professor Agnew, of Philadelphia, was an adjunct teacher for thirty years in anatomy before being made Professor of Surgery, and in him we have a noble exemplar for the whipper-snappers in swaddling clothes who form a large part of the teaching and controlling faculty in many medical colleges, and spout most vociferously in 'Varsity Vein' of higher medical education and long terms."

The third annual meeting of the American Academy of Railway Surgeons will be held in Chicago, Illinois, at the Auditorium on Wednesday, Thursday, and Friday, September 23, 24, and 25, 1896.

OFFICERS.

*President.*—John E. Owens, M.D., Chief Surgeon C. & N. W. Railway, Chicago, Ill.

*First Vice-President.*—L. E. Lemen, M.D., Division Surgeon U. P. Railway, Denver, Colo.

*Second Vice-President.*—F. L. Peck, M.D., Surgeon N. Y. P. & O. Railway, Clinton, N. Y.

*Secretary.*—Webb J. Kelly, M.D., Division Surgeon Erie Railway, Galion, Ohio.

*Treasurer.*— C. B. Kibler, M.D., Surgeon Erie Railway, Corry, Pa.

*Editor.*—R. Harvey Reed, M.D., Chief Surgeon C. S. &. H. Railway, Columbus, Ohio.

---

THE officers and graduates of Atlanta University are making an inquiry into the cause of the high death-rate among negroes in cities. The object of this inquiry is to secure the adoption of means for the improvement of the sanitary condition of the colored race in towns. This movement certainly deserves the cordial support of the friends of the negro.

The *Sanitarian* for June gives some alarming statistics on this subject, and it was this publication which moved the Atlanta University people to make the noble endeavor in which they are now engaged.

The *Sanitarian* shows that in Washington, where nearly one-third of the inhabitants are colored, the death-rates in 1895 are reported to have been as follows: White, 16.97; colored, 28.18.

For the disease commonly called consumption, the rates were : White, 1.77; colored, 3.98. The rate for the entire population was 20.57, and it will be observed that the rate for white people (16.97) is low for a city of this size. In New Orleans, where nearly one-quarter of the people are colored, the rates for the month of April were 23.66 and 51.45 respectively. For the white residents of Charleston the rate was 19.36, while for the colored people of that city it was 35.86. In Baltimore the rates for April were 18.18 and 35.05, and in twenty-seven towns of North Carolina, for the month of March they were 14.1 and 25.6.

The negro death-rate in Atlanta is much lower than that in some of the cities mentioned in this list, and lower than that of the lowest given above. Still it is higher considerably than the death-rate among the whites.—*Atlanta Journal.*

---

### OTITIS MEDIA.

Dr. Wm. Lincoln (*Cleveland Jour. of Med.*), in an able article on the complications of purulent otitis media, says: We should not "allow a purulent otitis media to progress to either a bad or a good result without attention. In other words, pus collection and discharge in and from the ear should receive not less and later, but greater and earlier attention than an abscess in other parts of the body, for the reason that it is less liable to get well without such attention, and also because the consequences of neglect are, in the general run of cases, more disastrous. In no class of cases does a patient suffer more or longer from inattention or from a mistaken conservatism of his physician."—*St. Louis Med. and Surg. Journal.*

may decline to publish any paper *not handed to the secretary complete* before the final adjournment of the annual meeting.

Dr. George Ben Johnson, 407 E. Grace street, Richmond, Va., is chairman of the committee of arrangements, who should be addressed in regard to hotel accommodations and railway fares.

Joseph Price, President.

William Warren Potter, Secretary.

---

Cæsarean Section.—Readers will be interested in the perusal of Dr. Allred's description (see p. 452) of his operation for Cæsarean Section; and whether or not they may concur in the choice of such procedure under the circumstances, they will certainly be inclined to heartily congratulate the patient and him on the successful issue of what must be looked upon as one of the most extraordinary abdominal sections on record.

---

### About Cocaine.

Following is a summary of an interesting paper on the "Rational Use of Cocaine in Surgery." (Dr. C. A. Dundore, *Medical and Surgical Reporter*):

1. The use of cocaine in surgery should not be abandoned because its irrational employment has produced deleterious results.
2. Always make a thorough physical examination of the patient before injecting the drug.
3. It should not be used in cases showing organic disease of the brain, heart, lungs, or kidneys, or in persons of a neurotic diathesis.
4. Children bear it fully as well as adults.
5. The patient should always be placed in a recumbent position prior to its employment.
6. Constriction should be used, whenever possible, to limit the action of the drug to the desired area.
7. Use a freshly prepared solution for each case.
8. Distilled water is to be employed, to which phenic, salicylic, or boric acid is to be added.

9. A two per cent. solution has a better effect and is safer than stronger solutions.

10. Never inject a larger quantity than 1⅛ grains when no constriction is used.

11. About the head, face, and neck, one-third grain may never be exceeded.

12. When constriction is possible, the dose should be as large as two grains.

13. Every slight physiological effect is not necessarily to be taken as cause for alarm.

14. Cocaine *does* have effect on inflamed tissues.

15. In case alarming symptoms threaten, use amyl nitrite, strych nine, digitalis, ether, or ammonia.

---

### INCOMPATIBLE WITH ANTIPYRIN.

According to the *Phar. Centralblatt* the following substances precipitate antipyrin from aqueous solution: (1) Phenic acid in concentrated solution; (2) tannin and tannic acid preparations; (3) tincture of iodine; (4) chlorides of mercury. The following decompose antipyrin when triturated with it in a dry state: (1) calomel, forming a toxic combination; (2) beta-naphthol; (3) chloral, which forms an oleaginous liquid; (4) bicarbonate of soda (an acetic ether odor is given off); (5) salicylate of soda, also forming an oleaginous liquid; (6) the salts of quinine and caffein, of which the solubility is increased by antipyrin.—*New York Medical Record.*

---

### SYPHILIS AND LOCOMOTOR ATAXIA.

Erb (*Berliner klinische Wochenschrift,* No. 11, 1896) makes a statistical report regarding the etiological bearing of syphilis on locomotor ataxia. He excludes women and persons of the lower classes, and reports 200 cases of tabes affecting men of the upper class, of whom 185—92½ per cent.—had a history of previous syphilitic infection; 61½ per cent. had positive secondary symptoms; 31 per cent. chancre without apparent secondary manifestations. Of the fifteen not infected, eleven had suspicious histories: repeated attacks of gonorrhea, strictures, buboes, miscarriage of wife, leukoplakia, syphilis of the father, etc. Of 200, only four were "absolutely now syphilitic," if one can so speak. Adding these 200 to a series of 500 previously reported, his statistics are, 90.35 per cent. of 900 cases of locomotor ataxia with syphilitic history, 9.65 per cent. without such history.—*University Med. Magazine*, June.

---

### DESCENDING OPTIC NEURITIS CONSEQUENT UPON NASAL TREATMENT.

Temporary amblyopia and narrowing of the field of vision, apparently reflex or combined with hyperemia of the eyeball, following the application of the galvano-cautery to the nasal mucous membrane, have been reported by a number of observers. A. Alt, St. Louis, reports (*American Journal of Ophthalmol.*, vol. xii., No. 9) a case of a somewhat different and more serious nature.

A man, aged thirty-eight years, had been treated for nasal catarrh and narrowing of the left nasal passage by means of different applications and the galvano-cautery. He noticed the left eye did not see so well as usual, and mentioned it to his physician. Treatment was persisted in, and two days later he awoke to find the eye blind. When seen that day by Alt there was doubtful perception of light, the pupil was dilated and scarcely reacting. The margins of the optic papilla were almost invisible, the retinal veins were hyperemic, the arteries thin. Tension was normal.

The nasal treatment was stopped. On the third day the patient counted fingers at one foot, and on the sixth day at five feet, with considerable increase of the field. A few days later injections of strychnine were commenced, and by the eighteenth day he had regained vision = 20/xxx+, the field being still somewhat narrowed except upward and outward. The arteries were still thin, and papilla almost white.

Afterward Dr. Alt learned that the patient had had syphilis, and he thinks that to this was in some measure due the severity of the attack; but that it could only be accused of being an indirect cause of the trouble by rendering the patient more vulnerable, since only the eye on the affected side was involved, and it improved promptly on mere cessation of the nasal treatment.—*American Journal of Medical Science*, May, 1896.

